# MLVA Genotyping Characteristics of Human *Brucella melitensis* Isolated from Ulanqab of Inner Mongolia, China

**DOI:** 10.3389/fmicb.2017.00006

**Published:** 2017-01-18

**Authors:** Zhi-Guo Liu, Dong-Dong Di, Miao Wang, Ri-Hong Liu, Hong-Yan Zhao, Dong-Ri Piao, Guo-Zhong Tian, Wei-Xing Fan, Hai Jiang, Bu-Yun Cui, Xian-Zhu Xia

**Affiliations:** ^1^College of Veterinary Medical Inner Mongolia Agriculture UniversityHohhot, China; ^2^Ulanqab Centre for Endemic Disease Prevention and Control, Health and Family Planning Commission of UlanqabJining, China; ^3^Laboratory of Zoonoses, China Animal Health and Epidemiology CenterMOA, Qingdao, China; ^4^State Key Laboratory for Infectious Disease Prevention and Control, Collaborative Innovation Center for Diagnosis and Treatment of Infectious Disease, National Institute for Communicable Disease Control and Prevention, Chinese Center for Disease Control and PreventionBeijing, China; ^5^Institute of Military Veterinary AMMSChangchun, China

**Keywords:** *Brucella melitensis*, MLVA, molecular epidemiology, trace-back analysis, Ulanqab, Inner Mongolia

## Abstract

Brucellosis is a serious public health problem in Ulanqab, which is a region located in the middle of the Inner Mongolia Autonomous Region adjacent to Shanxi and Hebei provinces. The disease is prevalent in both the latter provinces and Ulanqab with the highest prevalence of brucellosis occurring in Inner Mongolia. The MLVA-16 scheme is a genotyping tool for assessing genetic diversity and relationships among isolates. Moreover, this genotyping tool can also be applied to epidemiological trace-back investigations. This study reports the occurrence of at least two *B. melitensis* biovars (1 and 3) in Ulanqab, encompassing 22 and 94 isolates, respectively. *B. melitensis* biovar 3 was the predominant biovar in the area examined. Panel 1 (MLVA-8) identified three genotypes (42, 63, and 114), with genotype 42 (*n* = 101) representing 87% of the tested strains. MLVA-11 identified eight genotypes (116, 111, 297, 291, and 342–345) from 116 of the analyzed isolates. All of these isolates were identified as belonging to the East Mediterranean group. Genotype 116 (*n* = 94) was the predominant genotype and represented 81% of the isolates. The isolates pertaining to this genotype were distributed throughout most of Ulanqab and neighboring regions. The MLVA-16 scheme showed the presence of 69 genotypes, with 46 genotypes being represented by single isolates. This analysis revealed that Ulanqab brucellosis cases had epidemiologically unrelated and sporadic characteristics. The remaining 23 genotypes were shared (between a total of 70 isolates) with each genotype being represented by two to eight isolates. These data indicate that these cases were epidemiologically related. MLVA genotyping confirmed the occurrence of a multipoint outbreak epidemic and intrafamilial brucellosis. Extensive genotype-sharing events were observed among isolates from different regions of Ulanqab and from other provinces of China. These findings suggest either a lack of control of animal movement between different regions or the circulation of contaminated animal products in the market. Our study is the first comprehensive genotyping and genetic analysis of *B. melitensis* in Ulanqab. We believe that this study will help to improve the effectiveness of brucellosis control programs.

## Introduction

*Brucella* is a genus of Gram-negative facultative intracellular pathogens that infect humans and a variety of animal species (Shevtsov et al., 2015). Brucellosis is transmitted to humans following direct contact with infected animals or indirectly through the consumption of unpasteurized animal products (Deshmukh et al., 2015). The disease causes severe morbidity in humans and results in many medical challenges globally, especially in poor regions (Corbel, 1997; Pappas et al., 2006; Ducrotoy et al., 2015). Furthermore, animal brucellosis causes abortion and infertility in livestock, resulting in serious economic losses (Araj, 1999).

Since 2000, the incidence of brucellosis has increased rapidly in many regions in China (Deqiu et al., 2002; Jiang et al., 2011). The incidence of brucellosis in Inner Mongolia ranks in the top three incidence rates in Chinese provinces. The incidence of brucellosis in Ulanqab was 177.1/100,000 cases in 2011, 42.2/100,000 cases in 2012, 17.2/100,000 cases in 2013, 24.1/100,000 cases in 2014, and 26.5/100,000 cases in 2015. Ulanqab is a relatively small region in Inner Mongolia, covering a geographical area of 545,000 km^2^, and comprises 11 regions that are extensively pastoral and semi-pastoral. The people that inhabit these regions are economically dependent on small ruminant (sheep) livestock (Jiang et al., 2013). Livestock exchange occurs frequently in this region, and includes import from other areas, internal exchange, livestock foster care, and shifting field grazing. Due to excessive poverty and limitations in relation to control measures, brucellosis is endemic among sheep and humans in these regions. Technical difficulties have meant that previous studies only concentrated on *Brucella* infection surveillance in this region, and adequate attention was not paid to the molecular typing of *Brucella* species. However, rapid and accurate identification and typing procedures are important for epidemiologic surveillance, investigation of outbreaks, and control program follow-up (Whatmore et al., 2006; Al Dahouk et al., 2007; García-Yoldi et al., 2007). Previous studies have confirmed that MLVA (Multiple-locus variable-number tandem repeat analysis) is a useful tool for identifying and genotyping *Brucella* isolates and the resultant data can be used for genetic diversity analysis and epidemiology trace-back investigations (Whatmore et al., 2006; Kattar et al., 2008; Ferreira et al., 2012; Garofolo et al., 2013). In this study, the MLVA-16 scheme was used to type samples and determine the genotyping characteristics and epidemiological links of associated strains. The results of this study contribute to our understanding of the main transmission routes associated with this pathogen as well as effectively informing brucellosis control policies in Ulanqab.

## Materials and methods

### Ethics statement

This research was carried out according to the principles of the Declaration of Helsinki. The study is a retrospective investigation of historical strain collections using modern typing methods and the study protocol was approved by the Ethics Committees of the National Institute for Communicable Disease Control and Prevention and the Chinese Center for Disease Control and Prevention. Informed consent was obtained from all of the patients prior to diagnosis. *Brucella* spp. were isolated from patients' blood samples following confirmation of their consent.

### Bacterial strains

A total of 116 strains were examined. These strains were obtained from 11 Ulanqab counties and three neighboring regions [Suniteyouqi (Xilin Hot City of Inner Mongolia), Datong (Shanxi province), and Shangyi (Hebei province)] from 2011 to 2015. A total of 115 strains were recovered from 114 patients, and one strain was recovered from sheep. Two strains (ws091 and ws101) were obtained from the same patient. The *Brucella* strains were isolated and biotypes were identified using standard procedures (Alton et al., 1975; Al Dahouk et al., 2003). *B. melitensis* 16M, *B. abortus* 544, and *B. suis* 1330 reference strains were used as control strains. Species-level identification was undertaken using *B. abortus, B. melitensis, B. ovis, B. suis* PCR (AMOS-PCR; Bricker and Halling, 1994).

### DNA preparation

DNA was extracted with a Nucleic Acid Automatic Extraction System (LLXBIO China Ltd., China) using a single loop of fresh bacterial cells that were grown for 48 h on *Brucella* agar. DNA concentrations were measured by UV spectrophotometry (NanoDrop 2000, Thermo, US).

### *Brucella* MLVA-16 genotyping scheme

MLVA was performed as described previously (Le Flèche et al., 2006; Al Dahouk et al., 2007). The 16 primer pairs were divided into three groups: Panel 1 (MLVA-8: eight loci including bruce06, bruce08, bruce11, bruce12, bruce42, bruce43, bruce45, and bruce55), panel 2A (three loci including bruce18, bruce19, and bruce21), and panel 2B (five loci including bruce04, bruce07, bruce09, bruce16, and bruce30); MLVA-11 (panels 1 and 2A), and MLVA-16 (panels 1, 2A, and 2B). PCR amplifications were performed in 20 μL reaction volumes. The PCR conditions were as follows: initial denaturation at 94°C for 3 min, and then 30 cycles of 94°C for 30 s, 60°C for 30 s, and 72°C for 50 s, with a final extension of 72°C for 3 min. PCR products for the 16 loci were denatured and resolved by capillary electrophoresis on an ABI Prism 3130 automated fluorescent capillary DNA sequencer (Applied Biosystems). Fragments were sized following comparison with a ROX (carboxy-X-rhodamine)-labeled molecular ladder (MapMaker 1000; Bioventures Inc., Murfreesboro, TN, USA) and Gene Mapper software version 4.0 (Applied Biosystems). The fragment sizes were subsequently converted to repeat unit numbers using a published allele numbering system (Le Flèche et al., 2006; Jiang et al., 2013; Scholz and Vergnaud, 2013).

### Analysis of MLVA data

BioNumerics version 5.1 software (Applied Maths, Belgium) was used to analyze the MLVA-16 assay data. Both categorical coefficient and unweighted pair group methods were applied to clustering analysis. Polymorphisms at each locus were quantified using the Hunter-Gaston Diversity index, available on the HPA website (http://www.hpa-bioinformatics.org.uk/cgi-bin/DICI/DICI.pl; Hunter and Gaston, 1988). Resultant genotypes were compared using the web-based Brucella 2012 MLVA database. MLVA-11 was also used to determine phylogeographic relationships between our isolates and 528 isolates from MLVAbank including not available group (2), Africa group (4), America group (75), West Mediterranean group (77), China group (97), and East Mediterranean group (273). Minimum-spaning trees were constructed using the goeBURST algorithm with PHYOVIZ 2.0 (Francisco et al., 2012; Nascimento et al., 2017). MLVA-16 cluster analysis was extended to 194 *B. melitensis* strains including the Ulanqab isolates reported here. Seventy-eight strains from neighboring provinces of Inner Mongolia were utilized for the molecular epidemiology investigation. All MLVA profiles have been submitted to the MLVAbank (http://microbesgenotyping.i2bc.paris-saclay.fr/).

## Results

### Patient characteristics

Of the 114 *B. melitensis* isolates obtained from unique patients (two isolates were obtained from the same patient), 95% of the strains were isolated from individuals who were engaged in livestock farming and related work [including livestock traffickers (*n* = 3) and slaughterhouse workers (*n* = 1)]. The mean age of the 114 patients was 45.1 years (range 5–73 years), and the ratio of males (*n* = 79) to females (*n* = 35) was 2.26. The relative number of cases that exhibited pain, fever, fatigue, and sweating was 75.8, 46.6, 29.3, and 12.1%, respectively.

### Identification and distribution of *Brucella melitensis*

**Figure 1:**
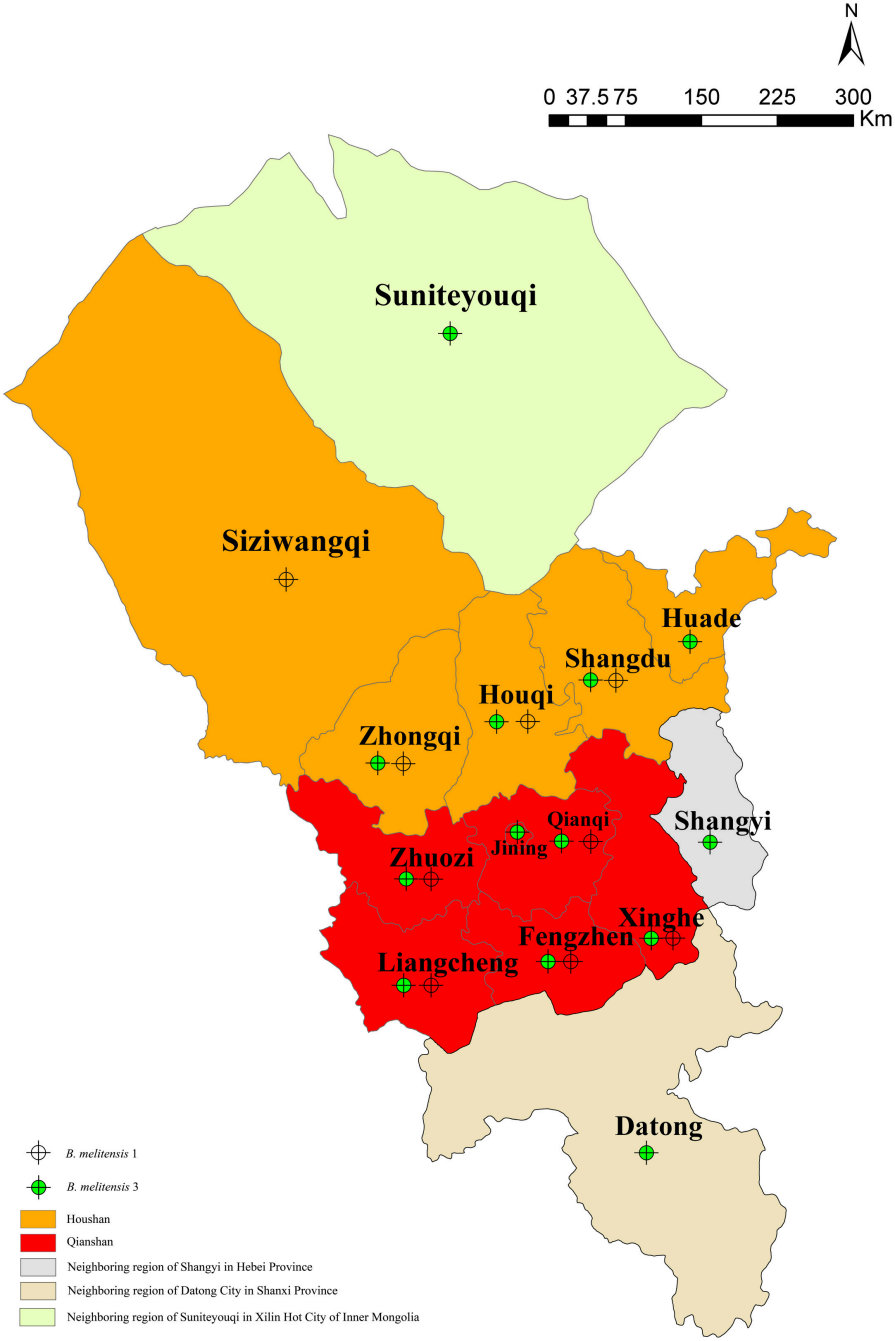
**Geographic distribution of ***Brucella*** samples in Ulanqab, Inner Mongolia, and neighboring regions Suniteyouqi (Xilin Hot City of Inner Mongolia), Shangyi (Hebei province), and Datong (Shanxi province)**.

All of the 116 *Brucella* strains were identified as *B. melitensis* bv. 3 (*n* = 94) and *B. melitensis* bv. 1 (*n* = 22) using the classical biotyping method. These strains were further examined by AMOS-PCR and 731 bp PCR products were obtained. Consistent with the classical biotyping approach, 116 *B. melitensis* strains were observed in the counties and surrounding areas of Ulanqab, including Suniteyouqi (1) (Xilin Hot City of Inner Mongolia), Datong (1) (Shanxi province), and Shangyi (3) (Hebei province) (Figure 1 and Table S1). Strains were predominantly distributed in the Qianshan (76 isolates) area of Ulanqab, especially, Liangcheng (22 isolates), Qianqi (20 isolates), and Fengzhen (18 isolates). An additional 30 strains (from the total of 116 isolates) were isolated from the Houshan area, with five strains being isolated from the areas surrounding Ulanqab. The original locations associated with the other five strains were unknown (Table S1).

### MLVA-16 genotyping results

In this study, results of a *B. melitensis* isolate diversity analysis confirmed the high discriminatory power of the MLVA-16 panel, with polymorphism levels of 0.985 based on the HGDI, compared to 0.235 and 0.337 for the MLVA-8 and MLVA-11 panels, respectively (Table 1). The HGDI in panel 1 was the highest (0.235) for bruce43. The bruce06, bruce08, bruce11, bruce12, bruce42, and bruce45 loci of panel 1, and bruce18 of panel 2A showed only one allele (HGDI = 0). In contrast, the greatest variability was detected in panel 2B (diversity index ≧ 0. 690) within loci bruce04, bruce16, and bruce30. The bruce04 locus displayed the highest diversity (HGDIs = 0.841; Table 1).

**Table 1:** **Allelic types and Hunter-Gaston diversity index (HGDI) of ***B. melitensis*** for 16 loci in this study**.

**Panel and Locus**	**Diversity**	**Index Confidence Interval**	**K**	**Max (pi)**
bruce06	0.000	0.000–0.061	1	1.000
bruce08	0.000	0.000–0.061	1	1.000
bruce11	0.000	0.000–0.061	1	1.000
bruce12	0.000	0.000–0.061	1	1.000
bruce42	0.000	0.000–0.061	1	1.000
bruce43	0.235	0.137–0.333	3	0.871
bruce45	0.000	0.000–0.061	1	1.000
bruce55	0.017	0.000–0.050	2	0.991
Bruce18	0.000	0.000–0.061	1	1.000
bruce19	0.115	0.038–0.193	3	0.940
bruce21	0.034	0.000–0.080	2	0.983
**Panel 2A**	0.147	0.061–0.233	4	0.922
bruce04	0.841	0.816–0.866	8	0.250
bruce07	0.084	0.015–0.153	3	0.957
bruce09	0.034	0.000–0.080	3	0.983
bruce16	0.791	0.748–0.834	11	0.328
bruce30	0.690	0.643–0.737	6	0.440
**Panel 2B**	0.983	0.976–0.989	62	0.069
**MLVA-16**	0.985	0.979–0.992	69	0.069
**MLVA-8**	0.235	0.137–0.333	3	0.871
**MLVA-11**	0.337	0.228–0.447	8	0.810

*Diversity Index (for VNTR data), A measure of the variation of the number of repeats at each locus. Ranges from 0.0 (no diversity) to 1.0 (complete diversity)*.

*Confidence Interval, Precision of the Diversity Index, expressed as 95% upper and lower boundaries*.

*K, Number of different repeats present at this locus in this sample set*.

*Max (pi), Fraction of samples that have the most frequent repeat number in this locus (range 0.0–1.0)*.

**Figure 2:**
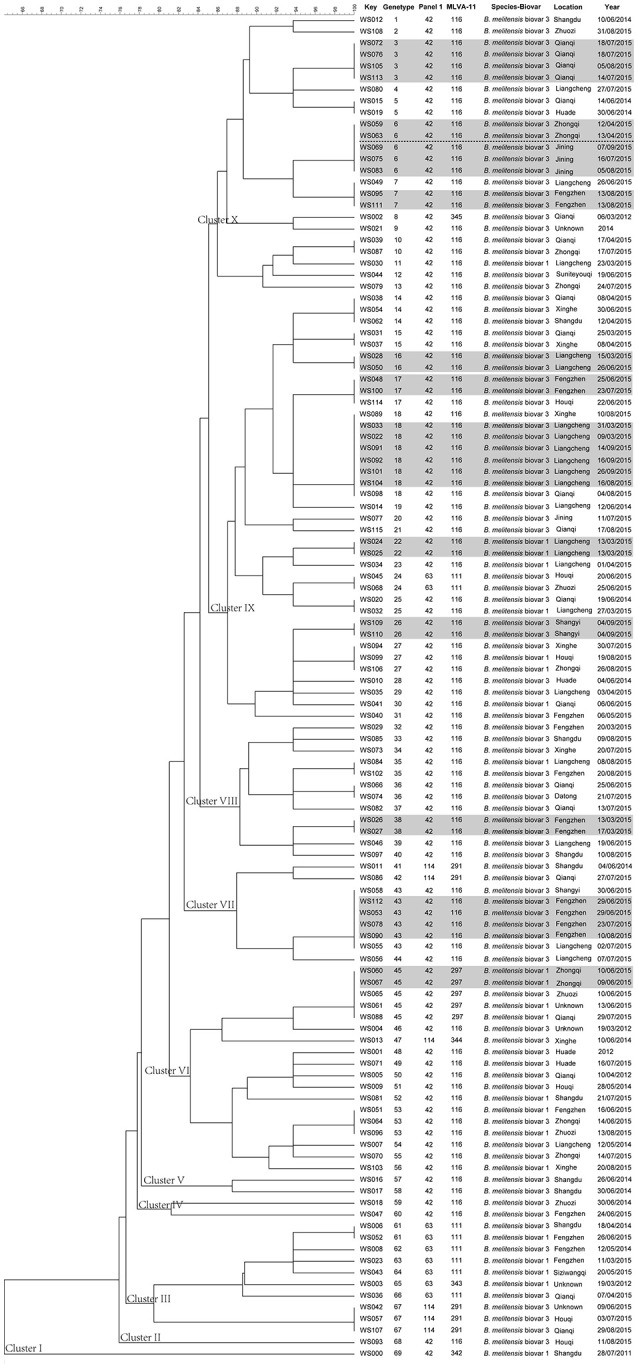
**Dendrogram based on the MLVA-16 genotyping assay (UPGMA method), showing relationships between the 116 ***B. melitensis*** isolates**. The columns show the identification numbers, MLVA-16 genotypes, panel 1 genotypes and MLVA-11 (panels 1 and 2A) genotypes, species-biovar, their geographic location, and the year of isolation of the strains.

Using the complete MLVA-16 scheme including panels 1, 2A, and 2B loci, 116 *B. melitensis* isolates clustered into 69 genotypes, 46 of which were represented by singular independent strains. The other 23 genotypes were shared by two or more isolates. Following analysis of data pertaining to panel 1, the *B. melitensis* population clustered into three known MLVA-8 genotypes: 42 (1-5-3-13-2-2-3-2; *n* = 101), 63 (1-5-3-13-2-3-3-2; *n* = 9), and 114 (1-5-3-13-2-1-3-2; *n* = 6). A dendrogram of the 116 *B. melitensis* strains shows strain identification features, the panel 1 genotype, panel 1+ panel 2A genotype, their geographical origins and the year of isolation (Figure 2). The clustering analysis showed that the 116 isolates formed ten main clusters. Clusters III and IX had two genotypes (114 and 63) and (42 and 63) each, respectively; Clusters VI and VII contained the same two genotypes (42 and 114); Clusters I, II, IV, V, VIII, and X all contained genotype 42.

**Figure 3:**
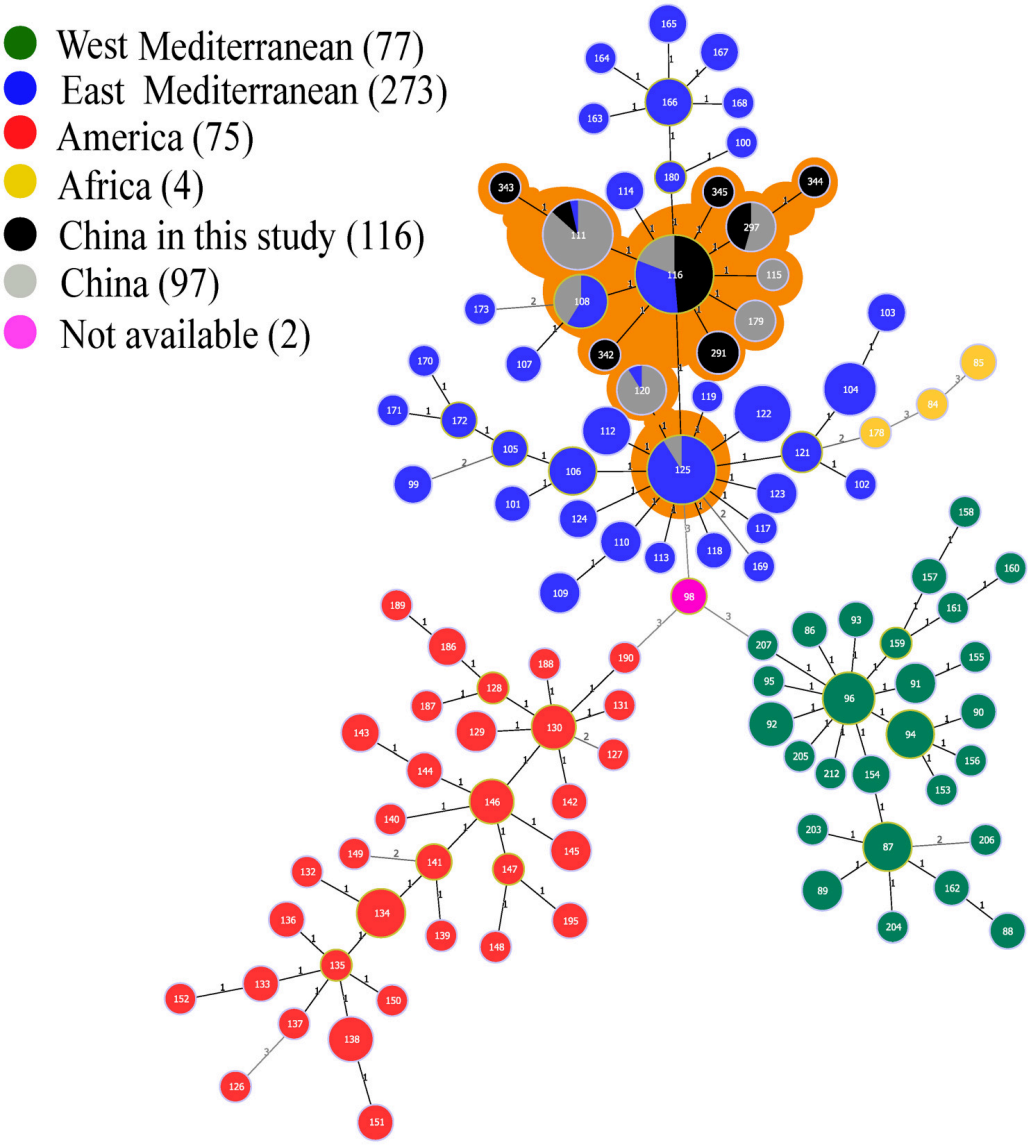
**Minimum spanning tree for ***B. melitensis*** using MLVA-11 data with the East Mediterranean group (blue), the America group (red), the Africa group (yellow), and the West Mediterranean group (blackish green), compared to the China genotypes (gray), and China isolates from our study (black)**. n.a. not available (pink). Samples with orange shadows indicate the China cluster within the East Mediterranean lineage.

The phylogeographic relationships of 116 Ulanqab *B. melitensis* isolates were assessed with MLVA-11 and eight different genotypes were identified. Four of these genotypes were previously described (116, 111, 297, and 291) and the remaining four novel genotypes represent single strains with single locus variants of these genotypes, and have been assigned numbers 342–345 in MLVAbank (http://microbesgenotyping.i2bc.paris-saclay.fr/). MLVA-11 genotypes 342 (1-5-3-13-2-2-3-2-4-36-8) and 345 (1-5-3-13-2-2-3-2-4-41-9) were single-locus variant (SLV) of MLVA-11 genotype 116 (1-5-3-13-2-2-3-2-4-41-8); MLVA-11 genotypes 343 (1-5-3-13-2-3-3-2-4-41-9) and 344 (1-5-3-13-2-1-3-2-4-46-8) were single-locus variants (SLV) of MLVA-11 genotypes 111 (1-5-3-13-2-3-3-2-4-41-8) and 297 (1-5-3-13-2-2-3-2-4-46-8), respectively. Genotype 116 was the predominant genotype, representing 81% (94/116) of the isolates. This genotype was broadly distributed throughout most of Ulanqab and neighboring regions. Minimum Spanning Tree analysis revealed that all of MLVA-11 genotypes are members of the “East Mediterranean” group (Figure 3 and Table S2).

### Cluster analysis for ulanqab *B. melitensis* genotypes

The MLVA-16 scheme indicated that there were 69 MLVA-16 genotypes (Figure 2), 46 of which were represented by unique strains. The other 23 were shared genotypes, with associated genotypes shared between two and eight isolates. The 23 shared genotypes were comprised of 70 clustered strains, with a clustering rate of 60.3% (70/116; Figure 2). The most frequently observed genotype, genotype 18, was present in eight isolates obtained from three regions over a 6-month period in 2015. The six strains that contained the second most frequently observed genotype, genotype 43, were isolated from three regions. Genotypes 6 and 45 were each present in five isolates and each genotype was present in two strains that were isolated from the Zhongqi.

### Relevance of MLVA-16 genotyping for clinical case and trace-back analysis of *B. melitensis*

**Figure 4:**
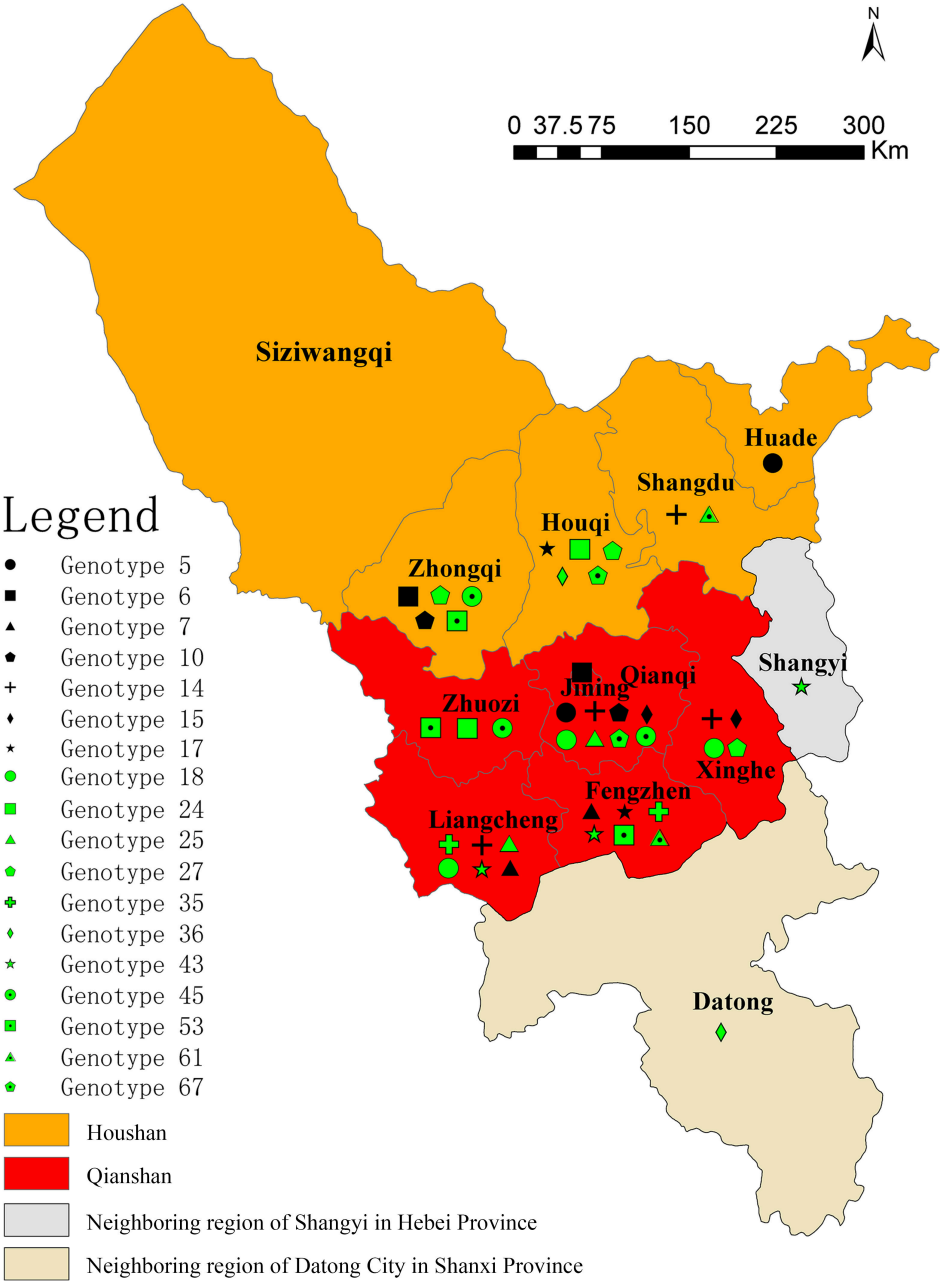
**District distribution of isolates with identical MLVA-16 genotypes**.

Eleven shared genotypes (Figure 2, genotypes with black shadow) were concurrently recovered from patients who came from the same area, two genotypes (22 and 26) of which were each recovered from patients of the same family. All associated patients contracted brucellosis from direct/indirect contact with infected sheep. Two isolates (genotype 7, ws049; and genotype 16, ws050) were obtained from members of the same family (mother and son) who engaged in sheep farming did not share the same MLVA-16 genotype. Two strains (ws091 and ws101) of genotype 18 were obtained from the same patient (13 days apart). Eighteen genotypes were shared between two or more isolates from two or more regions. The regions that exhibited shared genotypes are shown in Figure 4 and Table S3. All other isolates had distinct genotypes reflecting sporadic cases.

### Molecular epidemiological investigation of 194 chinese *B. melitensis* strains

Five *B. melitensis* strains were obtained from Xilin Hot City of Inner Mongolia (one strain), Hebei province (three strains), and Shanxi province (one strain). MLVA was used to determine the epidemiological links between 116 *Brucella* strains from Ulanpab (Table S1) and 78 *Brucella* strains from other provinces of China (Table S4). Ten genotypes were shared by strains from Ulanqab and 10 different provinces of China, including Inner Mongolia, Hebei, Shanxi, Shandong, Guangdong, Zhejiang, Liaoning, Xinjiang, Qinghai, and Beijing. Isolate BJ06-10 was isolated from a microbiology laboratory technician from Beijing hospital and was first reported in China by Jiang et al. (2011). This isolate contained an identical MLVA-16 pattern to isolates ws031 and ws037 from Ulanqab. Isolate ws097 exhibited an identical genotype to strains from four different provinces of China, Inner Mongolia, Shanxi, Hebei, and Guangdong, respectively. Furthermore, only the highest diversity locus (bru04) harbored alleles that were different from strains (TS-bruce051) isolated from Heilongjiang, China. As shown in Table S5, other strains from Ulanqab exhibited identical MLVA-16 profiles to strains isolated from different provinces of China.

## Discussion

In the present study, we used MLVA methods to genotype *Brucella* isolates from Ulanqab. A total of 116 *Brucella* isolates were examined by classical biotyping methods and MLVA typing. All isolates were *B. melitensis* (bv. 1 and bv. 3), and *B. melitensis* bv. 3 was the predominant biovar in this region. These data were in accordance with previous studies where human brucellosis was almost exclusively caused by *B. melitensis* bv. 3 in China (Deqiu et al., 2002). Furthermore, nearly all of the associated patients had a history of exposure to sheep. The results reveal that human brucellosis in Ulanqab is more related to infections in sheep than other livestock. Contact with infected domestic animals (sheep) has been identified in another study as the most likely source of infection (Hasanjani Roushan and Ebrahimpour, 2015). Since 2011, S2 vaccine immunization programs have been implemented for sheep for 5 consecutive years in Inner Mongolia. However, human brucellosis continues to occur with a considerable amount of *B. melitensis* infections diagnosed. Consequently, stringent animal quarantine measures could be a promising control strategy for human brucellosis in this region.

A total of 76 (65.5%) *B. melitensis* strains were isolated from the Qianshan area of Ulanqab. However, the incidence of brucellosis in Houshan, the region directly to the north of Ulanqab, is significantly higher than that in Qianshan districts. This may be related to the underreporting of cases in low-incidence areas. However, the most important reason relates to the fact that the Qianshan region is close to our hospital, and it is convenient for these individuals to attain a diagnosis and related treatment regimens.

Based on the MLVA-16 marker, our results clearly show that *B. melitensis*, despite the high genetic homogeneity within the genus, is highly polymorphic at the microsatellite level. The complete MLVA-16 profile offers excellent discriminatory power; the Hunter-Gaston index was 0.985. Panel 2B markers displayed very high discriminatory powers. This result is consistent with previous reports (Jiang et al., 2011; Xiao et al., 2015). With regard to MLVA-8 genotypes, the most frequently observed genotype in this study, genotype 42, was also observed in other regions of China (Jiang et al., 2011; Ma et al., 2016; Sun et al., 2016). MLVA-8 and MLVA-11 yielded three and eight genotypes, respectively; whereas the addition of panel 2B increased the number of genotypes to 69. These findings show that the genotypic variation of Ulanqab isolates was predominantly associated with the highly variable panel 2B loci and to a much lesser extent panel 2A (locus Bruce19 and 21) and panel 1 (loci Bruce 43) loci. In agreement with a previous report (Kilic et al., 2011), this may reflect microevolution through a stepwise mutational event of the most variable loci from a very limited number of ancestors. In addition, there was no clear relationship between biovar and genotype within this *B. melitensis* population. This finding is in agreement with previous studies that used multiple VNTR typing approaches to show that there was no discernible correlation between genotype and biovars (Whatmore et al., 2006). This suggests either that *B. melitensis* biovars do not adhere to specific genetic groups, or that biotyping is so subjective that field strains are often incorrectly assigned masking any potential genetic relationship (Whatmore et al., 2007).

The phylogeographic relationships of the 116 *B. melitensis* isolates from Ulanqab were compared to 528 MLVA profiles from the international database using MLVA-11. A minimum spanning tree analysis revealed the presence of eight different genotypes; all of the strains belonged to the East Mediterranean lineage. Distribution of the MLVA-11 genotypes was consistent in most of the provinces in China. Genotype 116 is the predominant genotype and is likely to have been historically present in this region. Moreover, this genotype was also responsible for the vast majority of *Brucella* infections in China. The four novel genotypes (342–345) represent single strains. Upon comparison with the MLVA international database we observed that the four novel MLVA-11 genotypes (342–345) had been observed for the first time in Ulanqab. No isolates from America and West Mediterranean lineages were observed in this study.

The MLVA-16 genotyping clustering rate was 60.3%, with most isolates differing in one to three loci in panel 2B. The proportion of strains being in clusters suggest that a significant proportion of brucellosis in Ulanqab is due to multiple contaminations from a single source. Four large clusters (genotypes 6, 18, 43, and 45) including strains from different regions. These data show that ongoing transmission of human brucellosis not only in a specific region but also among the regions in Ulanqab. Additionally, the determination that 46 isolates showed distinct genotypes reflected that more than 39.7% (46/116) of the brucellosis cases in Ulanqab had epidemiologically unrelated sporadic characteristics.

Previous studies have confirmed that MLVA-16 genotyping results show good correlation with epidemiological data with epidemiologically related isolates displaying identical or very closely related genotypes (Al Dahouk et al., 2007; Nöckler et al., 2009; Kilic et al., 2011; Allen et al., 2015) The current study also confirms the occurrence of multipoint outbreak epidemics and intrafamilial brucellosis. The 11 shared genotypes observed during this analysis pertained to 33 *Brucella* strains obtained from the same area. This suggested the occurrence of a multipoint outbreak epidemic from multiple common sources. Meantime, MLVA genotyping also confirmed intrafamilial brucellosis outbreaks in two cases (genotypes 22 and 26), where brucellosis most probably resulted from contact with infected sheep. Other nine shared genotypes confirms the occurrence of multipoint outbreak epidemics. This source of infection also resulted in a higher incidence of brucellosis in other regions of China (Xiao et al., 2015; Ma et al., 2016). In accordance with the previous studies (Kilic et al., 2011), two isolates (genotype 7, ws049; genotype 16, ws050) with different MLVA-16 genotypes were obtained from members of the same family are likely to have resulted from either persistent circulating strains causing sporadic infections or mutation events during the course of the outbreak.

Two isolates from human blood exhibiting identical genotypes were obtained 13 days apart from the same patient, suggesting the occurrence of a singular *B. melitensis* infection. This occurrence is consistent with previous research findings (Kasymbekov et al., 2013). These data confirmed that brucellosis bacteremia persist for at least 13 days (Memish et al., 2000; Pappas et al., 2006; Vrioni et al., 2008). The previous investigations confirmed that strains of low virulence (e.g., *Brucella abortus* S19) are almost always cleared from the bloodstream within a week, whereas virulent strains produce a bacteremia that regularly persists for 4 weeks and may last much longer (Cruickshank, 1957).

Eighteen shared genotypes (5, 6, 7, 10, 14, 15, 17, 18, 24, 25, 27, 35, 36, 43, 45, 53, 61, and 67) including the 58 clustered strains were obtained from two or more regions and displayed identical MLVA-16 profiles. MLVA-16 genotyping confirmed that more than half of the human brucellosis cases in this region resulted from either very close cross-transmission in a singular location or ongoing transmission among different regions. This is likely to have occurred as a result of the frequent trade and exchange of sheep in the associated regions (Garofolo et al., 2013; Shevtsov et al., 2015). In future, a more precise investigation of the strains circulating in animal reservoirs in these regions will be required to confirm this result.

When the strains observed during this analysis were compared with those from neighboring provinces of Inner Mongolia, 10 identical MLVA-16 genotypes were recorded, suggesting possible epidemiological links. Additionally, one strain was isolated from a microbiology laboratory technician in Beijing hospital in 2011 (Jiang et al., 2011). This isolate harbored an identical MLVA-16 profile with two isolates (ws031 and ws037) from Ulanqab. This result suggests that Ulanqab might be the source of infection. It should be noted that isolate ws097 from Ulanqab displayed an identical MLVA-16 profile with strains from four different provinces in China, Inner Mongolia, Shanxi, Hebei, and Guangdong, respectively. This result may be explained by the fact that Inner Mongolia is adjacent to northern Shanxi and Hebei province, and frequent livestock exchange occurs between these areas. These findings reflect either a lack of control in the movement of infected animals between regions or the circulation of infected animal products in the market. This suggests that inspection mechanisms and measures to ensure the quarantine of livestock exported from endemic regions should be more stringent.

In the present study, 116 *B. melitensis* isolates were genotyped and a comprehensive global genetic analysis was performed. All of the strains belong to the East Mediterranean lineage. We also observed that there was a broad genetic relationship among the analyzed population. Thus, we conclude that the MLVA-16 assay appears to be a very practical and important molecular genotyping tool that is capable of confirming epidemiological linkages in outbreak and trace-back investigations. This study will help to improve the effectiveness of brucellosis control programs.

## Author contributions

ZL performed most of the strain isolations and MLVA typing. ZL also drafted the manuscript; DD performed the MLVA cluster analysis and manuscript revision; MW and RL were in charge of epidemiological investigations and data analysis; HZ, GT, and DP prepared the DNA samples; HJ participated in the design of the study and critically reviewed the manuscript; BC, WF, and XX participated in the design of the study and also managed the project. All authors read and approved the final manuscript.

## Funding

This study was supported by the Medical and Hygiene Research Projects pertaining to the Inner Mongolia Health and Family Planning Commission (201301094), the National Nature Science Foundation (81271900), the Science and Technology Basic Work Program (2012FY111000), and the public welfare research special projects of Health and Family Planning Commission of China (201302006). The funders contributed to the study design and data collection.

### Conflict of interest statement

The authors declare that the research was conducted in the absence of any commercial or financial relationships that could be construed as a potential conflict of interest.
